# Sex-Specific Impact of Different Obesity/Metabolic Phenotypes on Long-Term Cardiovascular Outcomes in Acute Coronary Syndrome Patients

**DOI:** 10.3390/biomedicines10020424

**Published:** 2022-02-10

**Authors:** Egidio Imbalzano, Giuseppina T. Russo, Annalisa Giandalia, Angela Sciacqua, Luana Orlando, Vincenzo Russo, Maria Perticone, Arrigo F. G. Cicero, Antonio Giovanni Versace, Pierpaolo Di Micco, Vincenzo Antonio Ciconte, Giuseppe Dattilo, Giovanni Squadrito, Marco Vatrano

**Affiliations:** 1 Department of Clinical and Experimental Medicine, University of Messina, 98122 Messina, Italy; egidio.imbalzano@unime.it (E.I.); giuseppina.russo@unime.it (G.T.R.); luana_orlando@libero.it (L.O.); antonio.versace@polime.it (A.G.V.); giuseppe.dattilo@unime.it (G.D.); giovanni.squadrito@unime.it (G.S.); 2 Department of Medical and Surgical Sciences, University Magna Græcia of Catanzaro, 88100 Catanzaro, Italy; sciacqua@unicz.it (A.S.); mariaperticone@unicz.it (M.P.); 3 Department of Medical Translational Sciences, Division of Cardiology, Monaldi Hospital, University of Campania “Luigi Vanvitelli”, 80100 Naples, Italy; vincenzo.russo@unicampania.it; 4 IRCCS Policlinico S. Orsola—Malpighi, Hypertension and Cardiovascular Risk Research Center, DIMEC, University of Bologna, 40126 Bologna, Italy; arrigo.cicero@unibo.it; 5 Department of Medicine, Buonconsiglio Fatebenefratelli Hospital, 80100 Naples, Italy; pdimicco@libero.it; 6 UTIC and Cardiology, Hospital “Pugliese-Ciaccio” of Catanzaro, 88100 Catanzaro, Italy; vincenzo.ciconte@virgilio.it (V.A.C.); marco.vatrano1975@gmail.com (M.V.)

**Keywords:** sex, gender, acute coronary syndrome, metabolically healthy, obesity

## Abstract

Obesity, a major risk factor for acute coronary syndrome (ACS), is a multifaceted disease with different metabolic phenotypes and sex-specific features. Here, we evaluated the long-term cardiovascular risk by different obesity/metabolic phenotypes and by sex in ACS patients. The occurrence of the composite outcome of death, nonfatal reinfarction with or without PCI and/or stroke was evaluated in 674 patients (504 men; 170 women), consecutively hospitalized for ACS and followed-up for 7 years, who were stratified in metabolically healthy (MHNW) and unhealthy normal weight (MUNW), and in metabolically healthy (MHO) and unhealthy obese (MUO) groups. At baseline, 54.6% of patients were included in the MHNW group, 26.4% in the MUNW, 5.9% in the MHO and 13.1% in the MUO, with no sex-differences in the distribution of phenotypes. The overall rate of major outcome (100 person-years) in the reference group (MHNW) was higher in men than in women (RR: 1.19 vs. 0.6). The Kaplan–Meier curves for cumulative survival free from cardiovascular events according to obesity/metabolic status diverged significantly according to sex (log rank test, *p* = 0.006), this effect being more prominent in men (log 11.20; *p* = 0.011), than in women (log 7.98; *p* = 0.047). Compared to MHNW, the risk increased in obese men (RR: 2.2; 95% 1.11–1.54 in MUO group), whereas in women the risk was confined to metabolically unhealthy subjects (RR: 3.2; 95% CI 1.23–9.98, MUNW group). Our data show a sex-specific impact of obesity phenotypes on long-term cardiovascular risk in patients hospitalized for ACS.

## 1. Introduction

Obesity is a widely recognized risk factor for cardiovascular disease (CVD), morbidity and mortality [1]. Obesity also has adverse effects on metabolic factors, being a major component of metabolic syndrome (MetS) [2,3].

Current medical literature describes specific obesity phenotypes bearing different metabolic profiles and CVD risks. Thus, epidemiological studies have demonstrated that obesity free of metabolic abnormalities, i.e., individuals with the metabolic healthy obese (MHO) phenotype are at a lower CVD risk compared with metabolically unhealthy obese (MUO) ones [4]. However, not all data are concordant, and a recent study reported that the MHO phenotype may be associated with a higher risk of developing heart failure (HF), but not acute myocardial infarction (MI) [5].

Furthermore, an impaired metabolic profile associated with a normal weight was also demonstrated [4,5], suggesting the term of metabolically obese but normal weight (MONW) to describe individuals characterized by a higher susceptibility to type 2 diabetes (T2DM) and CVD, in spite of a normal weight [5]. This phenotype is not uncommon, and it seems to be associated with a high risk of all-cause and CVD mortality [5,6,7]. 

Up to date, the role of obesity on the CVD outcomes of subjects hospitalized for acute coronary syndrome (ACS) is still controversial, depending on the degree of obesity and on the length of follow-up, and data on the impact of different obesity phenotypes are even more sparse [8,9,10]. 

Moreover, sex-gender differences in obesity-related CVD outcomes may also play a role. Thus, obesity is more prevalent in women than in men worldwide, especially in low-income areas with a higher gender inequity gap [11,12]. Notably, sex/gender differences in the “obesity-lean paradox” on the risk of ACS recurrence have recently been demonstrated, with overweight ACS women showing the best prognosis [13]; furthermore, in ACS patients, significant interactions between gender-related variables and BMI on the 10-year prognosis were observed in females only [14].

These data suggest that cardiometabolic risk factors may be more strongly associated with adverse CVD outcomes than obesity per se [15], and that obesity-related phenotypes may differently impact the CVD risk in men and women. 

In order to better clarify these issues in a very high risk population, in this prospective study we evaluated the impact of four obesity/metabolic phenotypes on the risk of the composite outcome of death, fatal or nonfatal reinfarction with or without PCI and/or stroke in a large cohort of ACS patients observed for 7 years, taking potential sex differences into account.

## 2. Patients and Methods

### 2.1. Patients

A sample of 674 patients (504 men; 170 women) consecutively hospitalized for acute coronary syndrome (ACS) in the cardiology and clinical and experimental medicine departments of Messina University Hospital and Hospital “Pugliese-Ciaccio” of Catanzaro, Italy, from January 2011 to January 2013 and followed up for 7 years was included in the current analysis. Patients were eligible if they met the criteria for ACS, proposed by the Joint European Society of Cardiology/American College of Cardiology Committee, and if they underwent immediate coronary revascularization procedures (primary PCI) for ST-elevation myocardial infarction (STEMI) or coronary revascularization procedures (early PCI) within 24 h for myocardial infarction without ST-elevation (nSTEMI) or diagnostic coronary angiography following coronary artery bypass graft surgery (CABG). Exclusion criteria were recognized coronary artery disease or previous ACS, PCI following CABG, cardiogenic shock, atrial fibrillation, peripheral artery disease, severe cardiac valve disease and the presence of a prosthetic aorta. 

All participants were categorized into four groups, according to the presence or the absence of obesity and metabolic syndrome (MetS) as follows: metabolically healthy and normal weight (MHNW), metabolically unhealthy but normal weight (MUNW), metabolically healthy but obese (MHO) and metabolically unhealthy and obese (MUO). A written informed consent was obtained from each participant before initiating any study-related procedure. The study protocol was approved by the research review board of the participating hospital units.

### 2.2. Methods

#### 2.2.1. Blood Pressure Measurements

Clinical blood pressure (BP) readings were obtained in the supine position, after 5 min of quiet rest, with an aneroid sphygmomanometer. Systolic BP (SBP) and diastolic BP (DBP) were recorded at the first appearance (phase I) and the disappearance (phase V) of Korotkoff sounds, respectively. Baseline BP values were the average of three consecutive measurements obtained at intervals of 3 min. Patients with clinical SBP > 140 mmHg and/or DBP > 90 mmHg were defined as hypertensive [2].

#### 2.2.2. Body Mass Index

Height and weight were measured with participants wearing light clothes without shoes; height was measured to the nearest centimeter and weight was measured to the nearest half kilogram. BMI was calculated as body weight (kilograms) divided by the squared height (meters) and further subdivided into 2 categories: <30 kg/m^2^ (normal weight/nonobese overweight) and >30 kg/m^2^ (obese).

#### 2.2.3. Transthoracic Echocardiography (TTE)

Examinations were performed by two independent observers who were unaware of the clinical data, evaluated the recordings and calculated the parameters. Images were taken with the patient in the left decubitus position using VIVID 7 ultrasound machines (GE Technologies, Milwaukee, WI, USA) with an annular 2.5 MHz phased array transducer, as recommended by the American Society of Echocardiography [3].

#### 2.2.4. Metabolic Status

Waist circumference was measured at the height of the umbilicus, and hip circumference was measured at the thickest part of the hip (in only 442 patients). Fasting serum samples were analyzed for glucose, triglycerides and high-density lipoprotein cholesterol. The time since last meal (in hours) was recorded. Information on previously diagnosed diabetes mellitus and the use of blood pressure medication was collected from the self-administered questionnaire. We used a modified definition of metabolic health based on the MetS, as described by the International Diabetes Federation and MetS was defined according to an American Heart Association/National Heart, Lung, and Blood Institute Scientific Statement [16].

#### 2.2.5. Study Outcomes

The primary measure of outcome was a composite of death, fatal or nonfatal re-infarction with or without PCI and/or stroke occurring during the 7 years of observation. Each CVD outcome was included as a specific secondary endpoint. Procedure-related AMI within 24 h was not included into the endpoint. Study participants were followed from the coronary revascularization at entry, until they experienced one of the primary endpoints, proven by hospitalization. 

### 2.3. Statistical Analysis

Data are presented as means, standard deviations and frequency of occurrence (%). Continuous variables were compared with the paired or unpaired Student’s *t*-test, an ANOVA with a post hoc Tukey test, or with a simple Pearson correlation as appropriate. Discrete variables were compared using a Chi-squared analysis or a Fisher exact test. A test for normality was carried out on all variables using the Kolmogorov–Smirnov test, and non-normally distributed variables transformed for the purpose of the regression analysis. Furthermore, we calculated the relative risk ratio (RR) for CV events in patients with BMI ≥ 30 kg/m^2^ who were metabolically healthy or unhealthy, compared with normal-weight/nonobese overweight patients (BMI < 30 kg/m^2^) who were metabolically healthy (reference group). Incidence rates of CV recurrences were obtained by dividing the number of cases by person-years in each subgroup of metabolic phenotypes. Cox regression models were used to calculate the risk ratio between the incidence rate of CV events in metabolically healthy and unhealthy obesity subgroups divided by the incidence rate in the reference group. RR are presented as unadjusted, age- and sex-adjusted and multivariable-adjusted. Covariates in the multivariate-adjusted models were selected based on clinical relevance and when they increase >10% the risk ratio. Furthermore, the difference in rates of CV events among groups, according to metabolic status categorization, during the follow-up period was assessed by the Kaplan–Meier method by means of the log-rank test. A *p* value <0.05 was considered statistically significant. SPSS 20 statistical software was used for the analysis (Statistical Package for Social Sciences, Chicago, IL, USA).

## 3. Results

### 3.1. Baseline Clinical Characteristics According to Sex

Baseline clinical characteristics of the 674 subjects participating in the study (504 M, 170 F), according to sex and metabolic phenotypes are shown in Table 1. Overall, ACS women were older than men (68.8 vs. 62.4 years, *p* < 0.001) and they were less likely to be smokers (16.5 vs. 46%, *p* < 0.001). Hypertension and T2DM were more frequent among women. Systolic blood pressure (SBP) values were higher in women than in men (146 mmHg vs. 141.3 mmHg) whereas diastolic blood pressure (DBP) values were similar in the two groups. Heart rate was also higher in women than in men (75.8 vs. 70.5 beats/min), while echocardiographic parameters, including ejection fraction (EF, mean value 53.8%) and E/A ratio (mean value 0.3) were similar in the two genders at baseline. 

For the lipid profile, women had higher levels of HDL cholesterol (HDL-C) than men (48.6 vs. 43.7 mg/dL, *p* < 0.001); total cholesterol (181.7 mg/dL), LDL cholesterol (LDL-C; 107.0 mg/dL) and triglycerides levels (146.5 mg/dL) were not different in men and women. Women also presented higher levels of fasting blood glucose (FBG, 133.9 vs. 123.9 mg/dL, *p* = 0.058). Creatinine (1.0 mg/dL) and C-reactive protein (CRP) levels (10.5) did not differ in the two genders.

No sex differences were noted in cardiovascular therapies, including users of antiplatelet (99.5%), statin (99.3), diuretic (97.4%), ACE inhibitor (97.4%) and beta-blocker drugs, that were similar in ACS men and women.

Moreover, the extent of coronary artery disease (CAD), as assessed by the number of vessels affected by coronary disease (one- or two- or three-vessel disease), and the type of coronary revascularization procedure (single drug-eluting stent, multiple drug-eluting stents with overlapping, coronary artery bypass graft surgery) were comparable between men and women participating in the study. 

### 3.2. Baseline Clinical Characteristics According to Obesity-Related Phenotypes

Study subjects were stratified into four phenotypes, according to BMI and the presence of MetS. Of the 674 enrolled ACS patients, 368 subjects (54.6%), with baseline BMI values <30 kg/m^2^, were included in the MHNW subgroup, and 178 subjects (26.4%) in the MUNW. Among those with BMI values ≥30 kg/m^2^, 40 subjects (5,9%) were included in the MHO and 88 (13.1%) in the MUO group (Table 1).

Table 1 shows the baseline clinical characteristics analyzed according to obesity/metabolic phenotypes. No difference in age, echocardiographic parameters (EF and E/A), heart rate, type of coronary revascularization and pharmacological therapy were noted among the study groups. 

Overall, metabolic abnormalities were more represented in the MUNW group, followed by the MUO phenotype. Thus, the rate of T2DM (44.9%), SBP values (149.7 mmHg), HDL-C values (36.4 mg/dL), triglycerides (209.3 mg/dL), FBG (151.8 mg/dL) and smoking habit (42.1%; *p* = 0.062) were all significantly impaired in the MUNW subjects as compared with the other groups. The MUO phenotype was significantly associated with a higher rate of hypertension (86.4%) and abnormal values of DBP, HDL-C, triglycerides and FBG. Conversely, smoking habit was more frequent in the MHNW group (40.2%; *p* = 0.062), whereas total cholesterol and LDL-C, as well as creatinine and CRP mean levels were similar in the four groups.

The extent of coronary artery disease (CAD) also significantly differed among the obesity-related phenotypes, reflecting a gradual worsening of the disease from the metabolic healthy subjects to the unhealthy obese patients: subjects without significant CAD at baseline were more numerous in the MHO subgroup (20.8%) and poorly represented in the MUNW subgroup (2.8%); the highest rate of subjects with one-vessel and two-vessel diseases belonged to the MUNW group, and more than 38% of subjects of the MUO subgroup had a three-vessel disease (Table 1).

### 3.3. Baseline Clinical Characteristics According to Sex- and Obesity-Related Phenotypes

**Figure 1:**
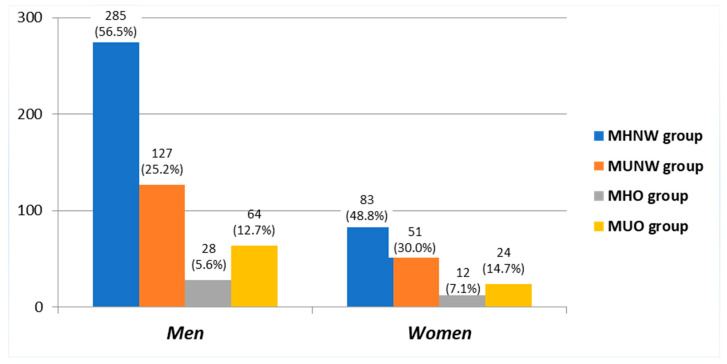
Baseline distribution of obesity-related phenotypes in men and women, separately. MHNW: metabolically healthy and normal weight; MUNW: metabolically unhealthy but normal weight; MHO: metabolically healthy but obese; MUO: metabolically unhealthy and obese. *p* < 0.001 for ANOVA comparisons among groups, both in men and women. *p* > 0.05 for Chi-square comparisons between men and women rates of obesity-related phenotypes.

Figure 1 shows the baseline distribution of obesity-related phenotypes in men and women, separately. Among the 170 women participating in the study, 83 (48.8%) were included in the MHNW subgroup, 51 (30%) in the MUNW group, 12 (7.05%) in the MHO subgroup and 25 (14.14%) in the MUO subgroup. Among the 504 men, 56.5% were included in the MHNW subgroup, 25.2% in the MUNW group, 5.5% in the MHO and 12.8% in the MUO group.

Clinical characteristics according to metabolic phenotypes were analyzed separately in ACS men and women (Table 2A,B). In both genders, no significant differences were noted with regard to age, creatinine, PCR, several echocardiographic parameters (ejection fraction and E/A), heart rate, type of coronary revascularization and pharmacological therapy across the different obesity-related phenotypes.

In men (Table 2A), metabolically unhealthy individuals (MUO and MUNW) showed the worst metabolic risk factor profile, especially the MUNW subgroup (Table 2A). Thus, smoking habit, diabetes, SBP, low HDL-C/High triglycerides and FBG were all significantly increased in the MUNW group, whereas the rate of hypertension and DBP values were higher in the MUO group (*p* < 0.01, all). Furthermore, male MUO subjects had the largest extension of CAD, with 39.1% of subjects with three-vessel disease at baseline (*p* = 0.007).

In ACS women (Table 2B), the distribution of major CVD risk factors among obesity-related phenotypes was similar to that observed in men. Thus, diabetes, lower HDL-C and higher triglycerides values, and higher FBG were also more frequent in the MUNW group than in the others, followed by the MUO phenotype for the frequency of major risk factors. However, in women, no differences in hypertension rate, as well as in the extent of CAD according to obesity phenotypes were noted. The single drug-eluting stent procedure was also more frequent in women with MUNW (54.9%; *p* = 0.045). 

### 3.4. Cumulative Incidence of the Composite Outcome of Death, Fatal or Nonfatal Reinfarction with or without PCI and/or Stroke in ACS Patients, According to Sex and Obesity/Metabolic Phenotypes

**Figure 2:**
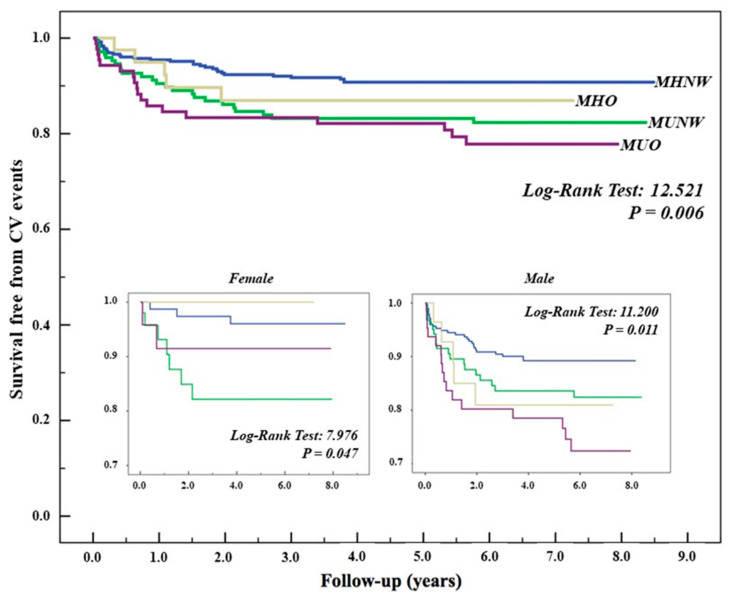
CVD risk according to metabolic phenotypes, overall and in men and women with ACS.

During the 7 years of observation, 80 CVD events including 4 CVD deaths, 69 nonfatal reinfarctions and 7 nonfatal strokes occurred. Figure 2 shows the Kaplan–Meier curves for cumulative survival free from CVD events (composite primary outcome of death, fatal or nonfatal reinfarction with or without PCI and/or stroke) stratified by BMI and metabolic status combining each other as previously described. As shown, the survival curves diverged significantly (log-rank test, *p* = 0.006) according to metabolic status rather than obesity, and this effect was more prominent in women (*p* = 0.047) than in men (*p* = 0.011).

The rate of CVD events (100 person-years) was 8.4% in the MHNW subgroup (reference group), 14.6% in the MUNW groups, 12.5 and 20.4 in the MHO and MUO groups, respectively. The univariate risk ratio (RR) adjusted for age and sex (Table 3) was 1.86 (1.06–3.24 95% CI) for the MUNW, 1.55 (0.56–4.25 95% CI) for the MHO and 2.79 (1.48–5.27) for the MUO subgroup, with the MHNW as the reference group. However, after multivariate adjustment for age, sex, smoking and total cholesterol, the RR was higher in the MUNW subgroup (RR 2.01; 1.19–3.38 95% CI), whereas it was 1.24 (0.77–1.99 95% CI) in the metabolic healthy obese (MHO) individuals and 1.38 (1.13–1.67 95% CI) in the metabolic unhealthy obese (MUO) subjects (Table 3).

The overall rate of CVD events (100 person-years) in the reference group (MHNW) was higher in men than in women (1.19 vs. 0.6), and it progressively increased across the obesity-related phenotypes (8.3 in the MUO group), but this trend was not observed in women (Table 4A,B).

As shown in Table 3 and in Figure 2, in the combined analysis, the overall risk of the composite primary outcome was highest in the MUO group (RR 2.79; IC: 1.48–5.27) with the univariate analysis, whereas, after multiple adjustment, the MUNW group showed the highest risk (RR: 2.01; IC: 1.19–3.38). A divergent trend was noted when this analysis was repeated in ACS male and female patients separately.

Thus, in men (Table 4A, Figure 2), the MUO group showed the highest RR both with the univariate (RR: 2.4; IC: 1.1–1.33) and multivariate analysis (RR: 2.2; IC: 1.1–1.54), followed by the MHO phenotype (RR: 1.39); conversely, in women, the MHO phenotype showed the lowest whereas the MUNW group showed the highest risk (RR: 1.8).

### 3.5. Factors Associated with the Incidence of the Composite Outcome of Death, Fatal or Nonfatal Reinfarction with or without PCI and/or Stroke in ACS Men and Women

Table 5 shows the factors significantly associated with the risk of CVD recurrence. In men, age, BMI, and HDL-C concentration were all significantly associated with the primary outcome, whereas these independent associations were not observed in ACS women. 

## 4. Discussion

Obesity affects millions of men and women worldwide, and BMI is linearly associated with CVD morbidity and mortality in both genders [2,17,18,19,20,21].

However, the role of different obese/metabolic phenotypes on the CVD risk in specific high-risk populations is still debated. Moreover, the effect of sex-gender differences has seldom been taken into account. Here, we demonstrated a different impact of obesity/metabolic phenotypes on long-term CVD outcomes in men and women presenting with ACS. 

Literature data reported better mortality outcomes in overweight or mildly obese patients with CVD, with an unexpected association between a higher BMI and survival that has been termed the “obesity paradox” [22].

Although all the reasons behind this paradox have not been fully clarified yet, they probably comprise the increasingly recognized “metabolically healthy obese” (MHO) phenotype, which includes obese patients without metabolic abnormalities typically associated with obesity.

In this study, we analyzed the predictive role of different obesity-related phenotypes, including obese subjects without MetS (MHO) and normal-weight patients with MetS (MUNW) on long-term CVD outcomes in a very high risk population, such as subjects hospitalized for ACS. In our cohort, the prevalence of MHO (5.9%) was similar in men and women, and comparable with that reported in other cohorts with different CVD risks [23,24,25]. Conversely, normal-weight subjects represented the vast majority of our ACS patients, with the MHNW group being the most represented group (54.6%), followed by the MUNW group (26.4%). In this setting, we identified a stronger impact of metabolic profile than the degree of obesity on the composite CVD outcome of death, fatal or nonfatal reinfarction with or without PCI and/or stroke, the risk being significantly higher in the metabolically unhealthy groups (MUO and MUNW). 

In accordance with our findings, several epidemiological studies have demonstrated a lower morbidity and mortality risk in MHO subjects when compared to MUO individuals, suggesting that cardiometabolic risk factors have a stronger impact than obesity per se on the CVD risk [23,24,25,26,27].

However, several lines of evidence indicate that MHO subjects may not be fully protected from a CVD risk, including heart failure [28]. An increased CVD risk in MHO individuals was also reported in the San Antonio Heart Study [6], and in several systematic reviews and meta-analyses, showing that MHO is not a benign condition, and the degree of the associated risk may vary according to the length of follow-up and the adopted therapeutic/preventive strategies [29,30,31,32,33].

Accordingly, a meta-analysis of 14 prospective studies [32] reported a higher >15-year CVD risk in both MHO (pooled RR 2.00; 95% CI 1.79–2.24) and MUNW individuals (RR 1.81; 95% CI 1.56–2.10) compared to normal-weight subjects.

These data suggest a crucial role of the length of follow-up in interpreting the risk associated with the MHO phenotype, and our data collected during a 7-year follow-up confirm the results of other long-term studies. Accordingly, it has been recently demonstrated that MHO subjects would change their phenotype over time. In this regard, the MESA study (Multi-Ethnic Study of Atherosclerosis) demonstrated that almost half of the participants would develop MetS during the follow-up over >12 years, and subjects with this “unstable” MHO phenotype would have a linear increase of the risk according to the length of the exposure to metabolic abnormalities [34]. Moreover, Fingeret et al. also recently reported that in a group of 179 MHO subjects, less than 50% remained MHO during follow-up, whereas the majority of subjects developed MetS factors [35]. 

When we analyzed the distribution of metabolic risk factors across BMI phenotypes in our study population, we found that the MUNW group carried the highest burden of risk factors, followed by the MUO subjects, as it was partly expected according to the study design, grouping patients by BMI and MetS status. 

Notably, the burden of T2DM was particularly high in the MHNW group, especially in men. In spite of the potential effect of the reduction of the sample size in each subgroup after stratification by sex and phenotypes, it is likely that in nonobese subjects, glucose metabolism derangement may be the major factor for developing ACS.

There is a complex relationship among insulin resistance, obesity and inflammation, in which inflammatory molecules and microRNAs (miRs) expression play an incisive role. Indeed, obese subjects overexpress inflammatory cytokines and oxidative stress markers, which could negatively affect the cardiac performance; some microRNAs (miRs) are key regulatory factors in lipid formation and lipoprotein synthesis and changes in microRNA profiles of various tissues correlate with obesity and MetS.

In women with and without T2DM, inflammatory markers correlated with more atherogenic lipid profiles, including specific HDL subpopulations [36]. Furthermore, it has been recently reported that obese subjects with prediabetes overexpressed inflammatory/oxidative stress molecules, miR-195 and miR-27, when compared to obese normoglycemic patients [37], suggesting that the relationship between obesity and inflammation may be particularly dangerous in subjects with altered glucose homeostasis.

It has also to be noted that fasting blood glucose levels were overall high in all the examined subgroups. This may be related to an underlying T2DM, but also to “stress hyperglycemia”, which may have an enormous impact on outcomes in patients hospitalized for ACS. Thus, the role of high glucose levels in determining CVD outcomes in ACS subjects is well demonstrated and a tight glycemic control has been described as affecting cardiac remodeling and CVD outcomes in ACS patients [38,39].

The risk category of the enrolled population is another important issue to be considered. ACS is a critical manifestation of CAD, carrying a high mortality and disability burden. MetS is associated with ACS in young patients (<45 years), where obesity is the most prevalent risk factor [40,41].

Studies on the role of obesity-related phenotypes in ACS patients are still limited [42,43].

Overall, obese patients with ACS appear to have more favorable short-term outcomes, although the benefits seem to disappear over time. Thus, in the MERLIN-TIMI 36 trial, on over 6000 ACS patients, those with BMI > 30 kg/m^2^ had a significant lower risk of the primary endpoint at 30 days, whereas no difference according to BMI was observed at 1 year [9]. A similar short-term benefit was observed in the BARI registry [44], whereas no differences according to BMI were observed in the short-term outcomes (30 days) of subjects with an overall lower baseline CVD risk, presenting to the emergency units with ACS-like symptoms [8].

In patients enrolled in our study, who underwent a baseline coronary revascularization for ACS, our results point to the predominant role of the metabolic milieu vs. obesity per se in the definition of the long-term CVD risk. Our findings are consistent with other large-scale studies indicating that metabolically healthy individuals, either with or without obesity, have a lower CVD risk than those with MetS [23,24,25], although not all the studies are concordant [6,30].

The type of outcome and/or potential age and sex differences may also contribute to explain the inconsistent results in the literature. Thus, a recent meta-analysis confirmed a higher risk for CVD and all-cause mortality among MHO individuals, with a significant effect of age and sex [45]. As for age, a nationwide analysis showed that overweight and obese elderly patients had a lower risk of mortality, CVD events and procedure-related complications [46]. 

In the hospitalized geriatric population participating to the REPOSI study, specific sex-aging phenotypes showed different mortality outcomes, with middle-aged men with multimorbidity and older women with severe cognitive decline being at higher risk [47]. 

In our study, women with ACS were older (68.8 vs. 62.4 years) and had more MetS risk factors than men; in this population we showed that the risk of CV events according to obesity-related phenotypes is also profoundly influenced by sex. 

It is now widely recognized that the risk of CVD events is profoundly different in men and women, due to multiple and multifaceted factors [48].

CVD risk factors may have a different burden in men and women, as reported for T2DM [49,50]. Moreover, obesity differently impacts men and women worldwide, because of genetic, ethnical, hormonal and social-related aspects [51], with clinical implications for the CVD risk. Thus, several studies have documented that obesity is significantly associated with the CVD risk in both men and women [9,42], but sex differences in morbidity and mortality have been reported in specific populations [52]. 

In particular, body characteristics and specific localization of fat deposition could influence the CVD risk in women, and the mammary gland has been reported to be a specific target for fat accumulation in premenopausal women. Indeed, in a large population of women aged >40 years in premenopause, a greater distribution of mammary fat and a lower breast density affected the incidence of MACEs at the 10-year follow-up [53].

Furthermore, it has been reported that the obesity paradox, i.e., the potential beneficial effect of the MHO phenotype on the CVD risk, seems to be more evident in women than in men [5,6,7]. In Swedish men, an increased CVD risk associated with MHO status has been demonstrated [5], whereas in the San Antonio Heart Study, the risk of developing CVD was found to be increased in MHO, regardless of sex [6]. 

In our study, we identified sex differences in the CVD risk associated with different obesity-related phenotypes, with obesity appearing to be a significant risk factor for the composite outcome in men but not in women. Among females, MHO participants had the least risk with no reported events, whereas women with the unhealthy phenotype (MUNW and MUO) showed a higher risk, irrespective of BMI; the opposite was true for obese men, since both obesity phenotypes (MHO and MUO) were at higher risk. 

Thus, our data demonstrated that the “overweight paradox” was more evident in ACS women than in men, since MHO women were the most protected against major events over a long follow-up, as also reported in other studies [14,54]. 

Several aspects merit considerations for the interpretation of our results. Women hospitalized for ACS were older, more obese and with an overall higher risk factors burden, including T2DM, when compared to men.

The older age in ACS women is an expected finding because of the ~10-year gap in the CVD risk due to estrogen effects during the reproductive age [51]. Furthermore, the larger prevalence of T2DM among women confirm the well-known greater impact of T2DM on the CVD risk in women than in men [55,56]. Thus, in women included in our study, it is likely that T2DM had a greater burden on the CVD risk than obesity per se. 

Similarly, a Portuguese registry reported that obesity, hypertension, diabetes, and dyslipidemia were all more prevalent among women admitted for ACS between 2002 and 2019, who were also less likely to present with typical symptoms and to be treated according to guidelines, thus exposing them to worst in-hospital outcomes and a higher mortality [57].

Further examining the differences between men and women in our population, the CVD risk was overall higher in men than in women, and men had a larger extension of CAD, with significant differences according to obesity/metabolic phenotypes. 

Conversely, our data do not confirm the gender gap in cardiovascular treatments, that has been reported in other studies [58], since the rate of revascularization procedures as well as the use of drugs with CVD benefit were comparable between the two genders.

Moreover, although obesity may induce a variety of structural and functional alterations in the myocardium, with some sex differences [59,60], no differences in EF% as well as in other echocardiographic parameters were observed between men and women in our study. 

Several potential limitations should also be acknowledged in our study. First, the observational nature of the study may have prevented us from excluding all potential confounders and to detect cause–effect relationships. Moreover, the classification of obesity, that was mainly based on BMI, since the waist-to-hip ratio was collected approximately in two thirds of the population, has to be taken into account. In addition, the low number of women included in the study compared with men, which reflects the real-world gender distribution in patients hospitalized for ACS, may have influenced the CVD event rate in some subgroups.

Information on several gender-related variables, such as the socioeconomic status, physical activity and nutrition were also not available, as well as HbA1c and fasting insulin levels, which could have been important to elucidate the role of insulin resistance and T2DM diagnosis and control on CVD outcomes, especially in women. Finally, the risk for indication bias could not be fully ruled out, due to the recruitment carried out in only two centers. 

## 5. Conclusions

In conclusion, our data show a sex-specific impact of obesity phenotypes on the long-term CVD and mortality risks in patients hospitalized for ACS. Thus, while MHO women seemed to be protected over time, in men, obesity had a stronger impact irrespective of metabolic status. These data emphasize the importance of assessing metabolic status and implementing systematic metabolic surveillance in patients undergoing coronary revascularization procedures, even if their weight is normal. If confirmed, this hypothesis may offer an attractive pathophysiological basis for the different CV risks observed in obese individuals, with important consequences on their personalized clinical management.

## Figures and Tables

**Table 1:** Study variables at baseline according to sex and metabolic phenotypes.

	TOTAL (674 pts)	MALE (504 pts)	FEMALE (170 pts)	*p* Value	MHNW (368 pts)	MUNW (178 pts)	MHO (40 pts)	MUO (88 pts)	*p* Value
Demographic Characteristics									
Age, (year)	64.0 ± 11.6	62.4 ± 11.4	68.8 ± 11.1	<0.001 **	64.1 ± 12.1	64.3 ± 11.7	63.2 ± 10.6	63.5 ± 10.2	0.924 ^#^
Cardiovascular risk factors									
Smokers, (%)	38.6	46.0	16.5	<0.001 *	40.2	42.1	35.0	26.1	0.062 *
Hypertension, (%)	65.7	62.5	75.3	0.002 *	58.4	71.3	62.5	86.4	<0.001 *
Diabetes mellitus, (%)	28.0	25.6	35.3	0.020 *	19.0	44.9	12.5	38.6	<0.001 *
Clinical parameters									
Systolic BP, (mmHg)	142.6 ± 23.3	141.3 ± 23.7	146.4 ± 21.4	0.022 **	139.0 ± 22.9	149.7 ± 23.6	133.6 ± 21.8	146.3 ± 20.6	<0.001 ^#^
Diastolic BP, (mmHg)	79.6 ± 13.1	79.5 ± 13.0	80.0 ± 13.6	0.676	78.2 ± 13.0	79.6 ± 12.4	81.2 ± 13.4	84.8 ± 13.6	0.001 ^#^
Heart rate, (beats/min)	71.9 ± 13.2	70.5 ± 12.8	75.8 ± 13.7	<0.001 **	71.3 ± 12.5	72.4 ± 13.8	69.8 ± 17.2	74.3 ± 12.9	0.320 ^#^
Ejection fraction, (%)	53.8 ± 11.1	53.3 ± 10.8	55.3 ± 12.0	0.142	54.3 ± 11.0	52.3 ± 11.9	56.5 ± 12.4	54.3 ± 8.2	0.316 ^#^
E/A	0.3 ± 0.4	0.4 ± 0.4	0.3 ± 0.5	0.493	0.2 ± 0.4	0.4 ± 0.4	0.1 ± 0.3	0.4 ± 0.5	0.09 ^#^
Extent of coronary artery disease at baseline									
No significant coronary artery disease, (%)	9.8	10.1	9.5	0.547 *	12.8	2.8	20.8	6.8	0.005 *
1-vessel disease, (%)	36.9	37.7	34.7	0.485 *	37.0	39.9	35.0	31.8	
2-vessel disease, (%)	24.5	25.0	22.4	0.480 *	24.2	27.0	20.0	22.7	
3-vessel disease, (%)	28.8	30.2	24.7	0.175 *	26.1	30.3	25.0	38.6	
Coronary revascularization at baseline									
Single drug-eluting stent, (%)	43.6	45.2	38.8	0.145 *	41.0	48.9	45.0	43.2	0.386 *
Multiple drug-eluting stents with overlapping, (%)	12.0	12.3	11.2	0.697 *	12.2	12.4	7.5	12.5	0.843 *
Coronary artery bypass graft surgery, (%)	10.5	11.1	8.8	0.402 *	9.8	11.2	10.0	12.5	0.568 *
Biochemical markers									
Total cholesterol, (mg/dL)	181.7 ± 44.1	180.3 ± 44.5	185.6 ± 42.7	0.174	179.5 ± 43.0	185.7 ± 43.5	184.6 ± 49.9	181.3 ± 47.0	0.461 ^#^
HDL cholesterol, (mg/dL)	44.9 ± 13.6	43.7 ± 12.9	48.6 ± 14.9	<0.001 **	49.0 ± 13.8	36.4 ± 8.9	52.0 ± 11.8	42.1 ± 12.5	<0.001 ^#^
LDL cholesterol, (mg/dL)	107.0 ± 39.6	106.8 ± 40.2	107.6 ± 37.7	0.821	108.1 ± 38.8	107.3 ± 40.7	107.4 ± 45.8	101.3 ± 37.6	0.565 ^#^
Triglyceride (mg/dL)	146.5 ± 90.3	150.1 ± 94.3	136.3 ± 76.8	0.088	111.7 ± 47.2	209.3 ± 104.9	107.0 ± 35.6	181.9 ± 123.7	<0.001 ^#^
Fasting glucose, (mg/dL)	126.4 ± 59.7	123.9 ± 56.5	133.9 ± 67.7	0.058	114.7 ± 59.1	151.8 ± 58.6	104.4 ± 45.0	134.2 ± 52.2	<0.001 ^#^
Creatinine, (mg/dL)	1.0 ± 0.8	1.0 ± 0.7	0.9 ± 0.8	0.299	1.0 ± 0.9	1.0 ± 0.8	0.9 ± 0.3	0.9 ± 0.4	0.746 ^#^
C-reactive protein, (mg/dL)	10.5 ± 21.0	10.4 ± 20.3	10.8 ± 23.6	0.879	8.5 ± 14.6	11.4 ± 25.2	12.4 ± 12.7	15.4 ± 30.4	0.280 ^#^
Medication following coronary revascularization									
Antiplatelet therapy, (%)	99.5	99.1	98.6	0.540 *	99.4	99.6	99.2	100.0	0.954 *
Statins, (%)	99.3	97.2	96.2	0.612 *	99.7	99.4	98.9	99.5	0.944 *
Diuretics, (%)	28.3	27.5	26.4	0.654 *	27.4	27.7	28.6	29.7	0.554 *
ACE inhibitor, (%)	97.4	97.2	96.2	0.167 *	97.6	96.6	98.1	97.3	0.898 *
Beta-blocker, (%)	95.5	95.3	94.4	0.343 *	95.2	95.1	96.5	95.3	0.789 *

Data are *n*, %, mean ± SD. pts: patients; MHNW: metabolically healthy and normal weight; MUNW: metabolically unhealthy but normal weight; MHO: metabolically healthy but obese; MUO: metabolically unhealthy and obese; * *p* values are for Chi-square; ** *p* values are for independent Student t; ^#^ *p* values are for ANOVA.

**Table 2:** (**A**) Study variables at baseline according to metabolic phenotypes in men with ACS. (**B**) Study variables at baseline according to metabolic phenotypes in women with ACS.

**(A)**	**MHNW** **(285 pts)**	**MUNW** **(127 pts)**	**MHO** **(28 pts)**	**MUO** **(64 pts)**	***p* Value**
Demographic characteristics					
Age, (year)	62.9 ± 11.9	61.9 ± 11.1	60.9 ± 10.7	62.5 ± 9.8	0.765 ^#^
Cardiovascular risk factors					
Smokers, (%)	46.7	52.1	46.4	31.3	0.058 *
Hypertension, (%)	55.4	68.5	53.6	85.9	<0.001 *
Diabetes mellitus, (%)	17.5	40.9	7.1	39.1	<0.001 *
Clinical parameters					
Systolic BP, (mmHg)	136.8 ± 22.9	150.6 ± 23.9	131.1 ± 24.0	146.5 ± 19.1	<0.001 ^#^
Diastolic BP, (mmHg)	77.7 ± 12.9	81.3 ± 12.0	79.1 ± 12.9	84.2 ± 13.7	0.003 ^#^
Heart rate, (beats/min)	69.9 ± 12.0	71.1 ± 12.9	69.2 ± 18.6	72.9 ± 13.1	0.511 ^#^
Ejection fraction, (%)	53.7 ± 10.9	52.5 ± 11.7	54.6 ± 11.5	53.6 ± 7.3	0.829 ^#^
E/A	0.3 ± 0.4	0.4 ± 0.4	0.1 ± 0.3	0.4 ± 0.5	0.113 ^#^
Extent of coronary artery disease at baseline					
No significant coronary artery disease, (%)	9.5	1.6	14.3	4.7	0.007 *
1-vessel disease, (%)	38.6	37.1	46.4	31.3	
2-vessel disease, (%)	24.2	27.6	21.4	25.1	
3-vessel disease, (%)	27.7	33.9	17.9	39.1	
Single drug-eluting stent, (%)	43.5	46.5	50.1	48.4	0.817 *
Multiple drug-eluting stents with overlapping, (%)	13.7	10.2	7.9	10.9	0.759 *
Coronary artery bypass graft surgery, (%)	10.2	13.4	7.1	12.5	0.787 *
Biochemical markers					
Total cholesterol, (mg/dL)	178.9 ± 43.1	183.9 ± 44.7	178.5 ± 49.1	180.6 ± 48.8	0.753 ^#^
HDL cholesterol, (mg/dL)	47.6 ± 13.3	35.3 ± 8.6	48.9 ± 11.0	41.2 ± 10.0	<0.001 ^#^
LDL cholesterol, (mg/dL)	108.9 ± 39.4	105.8 ± 41.6	107.7 ± 46.6	99.2 ± 38.5	0.379 ^#^
Triglyceride (mg/dL)	113.4 ± 49.2	217.1 ± 104.4	108.2 ± 39.3	198.1 ± 139.2	<0.001 ^#^
Fasting glucose, (mg/dL)	115.1 ± 60.9	145.8 ± 50.8	100.5 ± 31.3	130.1 ± 40.7	<0.001 ^#^
Creatinine, (mg/dL)	1.0 ± 0.9	1.0 ± 0.7	0.9 ± 0.3	1.0 ± 0.4	0.905 ^#^
C-reactive protein, (mg/dL)	9.4 ± 15.5	9.7 ± 21.9	12.4 ± 12.7	16.1 ± 35.5	0.440 ^#^
Medication following coronary revascularization					
Antiplatelet therapy, (%)	98.4	97.6	97.1	99.9	0.564 *
Statins, (%)	99.3	99.1	97.3	98.8	0.173 *
Diuretics, (%)	28.4	27.9	29.4	29.1	0.223 *
ACE inhibitor, (%)	96.5	97.6	98.6	98.2	0.431 *
Beta-blocker, (%)	94.6	96.1	97.1	96.1	0.551 *
**(B)**	**MHNW** **(83 pts)**	**MUNW** **(51 pts)**	**MHO** **(12 pts)**	**MUO** **(24 pts)**	***p* Value**
Demographic characteristics					
Age, (year)	68.5 ± 11.6	70.4 ± 11.1	68.1 ± 8.1	11.1 ± 10.9	0.549 ^#^
Cardiovascular risk factors					
Smokers, (%)	18.1	17.6	8.3	16.0	0.792 *
Hypertension, (%)	68.7	78.4	83.3	84.1	0.210 *
Diabetes mellitus, (%)	24.1	54.9	25.1	36.1	0.003 *
Clinical parameters					
Systolic BP, (mmHg)	146.7 ± 21.2	147.5 ± 22.8	140.2 ± 14.3	146.1 ± 23.7	0.839 ^#^
Diastolic BP, (mmHg)	80.1 ± 13.4	75.5 ± 12.4	86.4 ± 13.7	86.1 ± 13.7	0.007 ^#^
Heart rate, (beats/min)	75.9 ± 12.9	75.9 ± 15.5	71.6 ± 14.1	77.1 ± 12.4	0.841 ^#^
Ejection fraction, (%)	56.9 ± 11.4	51.9 ± 12.5	63.1 ± 15.3	55.8 ± 10.5	0.191 ^#^
E/A	0.3 ± 0.5	0.4 ± 0.5	0.3 ± 0.4	0.7 ± 0.4	0.391 ^#^
Extent of coronary artery disease at baseline					
No significant coronary artery disease, (%)	24.1	5.9	33.3	12.1	0.367 *
1-vessel disease, (%)	31.1	47.1	8.3	32.0	
2-vessel disease, (%)	24.1	25.5	16.7	20.1	
3-vessel disease, (%)	20.5	21.6	41.7	36.1	
Single drug-eluting stent, (%)	32.5	54.9	33.3	32.0	0.045 *
Multiple drug-eluting stents with overlapping, (%)	7.2	17.6	0.0	16.1	0.129 *
Coronary artery bypass graft surgery, (%)	8.4	5.9	0.0	12.1	0.603 *
Biochemical markers					
Total cholesterol, (mg/dL)	181.6 ± 42.6	190.1 ± 40.7	199.1 ± 50.8	183.4 ± 42.8	0.476 ^#^
HDL cholesterol, (mg/dL)	54.2 ± 14.0	39.3 ± 9.2	60.3 ± 10.1	45.1 ± 17.7	<0.001 ^#^
LDL cholesterol, (mg/dL)	105.4 ± 37.1	111.2 ± 38.5	106.6 ± 45.9	107.5 ± 35.3	0.869 ^#^
Triglyceride (mg/dL)	106.1 ± 39.4	192.1 ± 104.8	104.1 ± 26.5	138.6 ± 52.8	<0.001 ^#^
Fasting glucose, (mg/dL)	113.4 ± 53.0	166.9 ± 73.1	113.6 ± 68.1	145.3 ± 73.8	<0.001 ^#^
Creatinine, (mg/dL)	0.9 ± 0.8	1.1 ± 1.0	0.8 ± 0.1	0.8 ± 0.2	0.513 ^#^
C-reactive protein, (mg/dL)	5.1 ± 12.6	16.1 ± 33.3	11.6 ± 22.1	13.9 ± 15.8	0.194 ^#^
Medication following coronary revascularization					
Antiplatelet therapy, (%)	99.3	96.4	95.9	98.4	0.453 *
Statins, (%)	98.2	98.4	97.3	99.3	0.226 *
Diuretics, (%)	30.1	28.6	28.5	29.6	0.331 *
ACE inhibitor, (%)	97.4	96.4	97.8	99.4	0.546 *
Beta-blocker, (%)	95.9	97.1	98.2	97.3	0.662 *

(A) Data are *n*, %, mean ± SD. pts: patients; MHNW: metabolically healthy and normal weight; MUNW: metabolically unhealthy but normal weight; MHO: metabolically healthy but obese; MUO: metabolically unhealthy and obese; * *p* values are for Chi-square; ^#^ *p* values are for ANOVA. (B) Data are *n*, %, mean ± SD. pts: patients; MHNW: metabolically healthy and normal weight; MUNW: metabolically unhealthy but normal weight; MHO: metabolically healthy but obese; MUO: metabolically unhealthy and obese; * *p* values are for Chi-square; ^#^ *p* values are for ANOVA.

**Table 3:** CVD recurrence according to metabolic phenotypes.

	MHNW	MUNW	MHO	MUO
Number of person-years	1951.05	780.95	207.30	438.87
Rate of CVD events (100 person-years)	8.4	14.6	12.5	20.4
Univariate risk ratio ^a^ (95% CI)	1.0	1.86 (1.06–3.24) ^c^	1.55 (0.56–4.25) ^c^	2.79 (1.48–5.27) ^c^
Multivariate risk ratio ^b^ (95% CI)	1.0	2.01 (1.19–3.38) ^c^	1.24 (0.77–1.99) ^c^	1.38 (1.13–1.67) ^c^

^a^ Adjusted for age and sex. ^b^ Adjusted for age, sex, smoking and total cholesterol. ^c^ Matched with MHNW population.

**Table 4:** (**A**) CVD recurrence according to metabolic phenotypes in men with ACS. (**B**) CVD recurrence according to metabolic phenotypes in women with ACS.

**(A)**	**MHNW**	**MUNW**	**MHO**	**MUO**
Number of person-years	1471.39	574.39	132.35	299.16
Rate of CVD events (100 person-years)	1.9	3.3	3.7	8.3
Univariate risk ratio (95% CI)	1.0	1.17 (0.66–2.08) ^c^	1.45 (0.52–3.88) ^c^	2.4 (1.01–1.33) ^c^
Multivariate risk ratio ^a^ (95% CI)	1.0	1.11 (0.71–1.98) ^c^	1.39 (0.61–3.11) ^c^	2.2 (1.11–1.54) ^c^
**(B)**	**MHNW**	**MUNW**	**MHO**	**MUO**
Number of person-years	479.66	206.56	74.94	139.90
Rate of CVD events (100 person-years)	0.6	3.4	0.0	2.1
Univariate risk ratio (95% CI)	1.0	3.6 (1.09–12.03) ^c^	0.9 (0.88–0.96) ^c^	2.1 (0.51–8.21) ^c^
Multivariate risk ratio ^b^ (95% CI)	1.0	3.2 (1.23–9.98)	0.0	1.8 (0.49–7.44) ^c^

(A) ^a^ Adjusted for age, smoking and total cholesterol. ^c^ Matched with MHNW population. (B) ^b^ Adjusted for age, smoking and total cholesterol. ^c^ Matched with MHNW population.

**Table 5:** Factors associated with CVD recurrence in men and women.

	TOTAL Exp(B)	*p* Value *	Male Exp(B)	*p* Value *	Female Exp(B)	*p* Value *
Male sex	0.524	0.425	-		-	
Age	1.054	0.119	1.102	0.019	0.566	0.997
BMI	1.216	0.022	1.251	0.038	1.169	0.667
Systolic Blood Pressure	0.973	0.122	0.958	0.141	0.777	0.563
Diastolic Blood Pressure	1.049	0.113	1.044	0.179	1.322	0.543
Heart rate	0.975	0.207	0.954	0.061	0.877	0.876
Ejection fraction	1.022	0.465	1.061	0.116	1.121	0.121
E/A	2.266	0.145	1.397	0.609	1.875	0.512
Total Cholesterol	1.015	0.369	1.020	0.269	0.987	0.867
HDL Cholesterol	0.973	0.332	0.905	0.023	0.676	0.771
LDL Cholesterol	0.983	0.313	0.982	0.330	0.232	0.911
Triglycerides	1.002	0.088	1.002	0.128	0.991	0.879
Fasting glucose	0.998	0.720	0.987	0.076	1.02	0.783
Creatinine	0.368	0.295	0.345	0.285	0.451	0.913
C reactive protein	1.000	0.978	1.007	0.614	0.999	0.985

* *p* values are for Logistic regression analysis.

## Data Availability

The datasets used and/or analyzed during the current study are available from the corresponding author on reasonable request.
